# Comprehensibility and impact of vehicle dashboard indicator light symbols on drivers’ preventive maintenance compliance

**DOI:** 10.1371/journal.pone.0323386

**Published:** 2025-05-14

**Authors:** Charles Atombo, Maxwell Selase Akple, Arnold Dumenya, Paul Adzimahe

**Affiliations:** 1 Department of Mechanical Engineering, Ho Technical University, Ho, Ghana; CREA: Consiglio per la ricerca in agricoltura e l’analisi dell’economia agraria, ITALY

## Abstract

**Background:**

Dashboard indicator light symbols convey information about vehicles status and functioning, but action taken to address issues associated with these symbols depend on drivers’ familiarity and comprehension of the symbols as well as demographic factors. Thus, this study assesses drivers’ familiarity and comprehension of dashboard indicator light symbols and further examines the impact of driver background factors and comprehension level of the symbols on drivers’ preventive maintenance compliance.

**Method:**

Through a survey involving 2530 drivers, familiarity and comprehension of 25 dashboard indicator symbols used on most vehicles were collected. Descriptive analysis and Generalised Ordered Logistic Regression were used to analyse the data.

**Results:**

Indicator light symbols that recorded a higher percentage of familiarity and were deemed appropriate were comprehended by most drivers. Symbols with pictorial signs were better understood. Overall, there were substantial significant differences among symbols in drivers’ familiarity and comprehension. Regression analysis indicates that the majority of dashboard indicators with low comprehension had a positively significant relationship, while symbols with better understanding recorded a negative relationship with driver’s preventive maintenance compliance. However, symbols such as tyre pressure, traction, 4*4 active, and power steering warning lights, which were poorly comprehended, showed a negative significant association with preventive maintenance compliance.

**Conclusion:**

The complex relationship between comprehension and drivers’ preventive maintenance compliance underscores the importance of including driver education programs and symbols standardisation to improve comprehension and preventive maintenance compliance, promoting road safety and vehicle reliability.

## 1. Introduction

Technological advancements have significantly transformed vehicles, especially in how they transmit information to drivers. One of the components of this evolution is the vehicle dashboard, which has developed from basic speed monitoring functions to sophisticated systems capable of providing comprehensive information about vehicle status and enhancing driver interaction [1,2]. The vehicle dashboard serves as a control panel and contains information, offering drivers and passengers real time information and alerts about the condition of the vehicle components. These communications are primarily delivered through indicators that use pictographic or pictorial (symbols) or text (Acronym) to guide drivers during operation [3].

The vehicle dashboard is equipped with indicator lights that are essential for safe and efficient vehicle operation. These lights are designed to capture the driver’s attention and prompt immediate action, when necessary, while also encouraging regular vehicle maintenance and safety practices [2]. Some dashboard indicators alert the driver as soon as the ignition is switched on and remain illuminated until the issue they signify is addressed. The International Standards Organization (ISO) has standardised dashboard symbols to provide important information about the vehicle’s operational status or system malfunctions. These symbols follow universal colour codes that are language independent for each vehicle make and model, allowing drivers across regions to understand them [3,4]. The strategic use of colours (yellow, orange, and red) indicates different levels of urgency, guiding drivers on when immediate action is required.

A study has indicated that certain dashboard lights, like the seatbelt indicator, remind drivers and passengers to fasten their seatbelts, promoting safety and compliance with regulations [5]. Similarly, symbols for airbags and anti-lock brakes inform drivers about the operational status of these important safety features, ensuring that they are functioning correctly [6]. Warning lights (orange or yellow in colour) serve as early warning systems that alert drivers on the vehicle status which require attention, though immediate action is not always necessary. This encourages drivers to engage in proactive maintenance practices or inspections to prevent more severe mechanical problems. For instance, a low tyre pressure prompts drivers to check and inflate tyres, which can prevent blowout and maintain optimal vehicle performance and safety. Emergency/advisories lights usually red in colour indicate serious problems that require the driver to stop the vehicle and check immediately to prevent damage or ensure safety. This immediate response can avert potential accidents or severe mechanical failures. For instance, a flashing oil pressure light indicates a serious problem with the engine’s oil system. It signifies that the oil pressure has dropped to a dangerously low level and ignoring this warning can lead to severe consequences, including engine failure and costly repairs. A study has emphasised that immediate action is necessary when this light illuminates to prevent serious engine damage [7].

When a warning light appears on the dashboard, an uninformed driver may panic, even if the issue is something minor. Therefore, A proper understanding of dashboard symbols can significantly reduce driver anxiety and increase confidence, which may help drivers respond appropriately instead of stressing unnecessarily. This is supported by a previous study which indicated that familiarity with the implications of these lights allows drivers to respond appropriately without undue panic [8]. This knowledge empowers drivers to handle vehicle issues more effectively, contributing to a smoother and more confident driving experience. For instance, a study has shown that a driver knowing that a yellow “check engine” light indicates a non-critical issue can help the drivers to address problems at a convenient time, minimising stress [9].

The layout and arrangement of dashboard indicator lights vary across vehicle makes and models, with manufacturers continuously evolving instrument panels to accommodate new technologies. These advancements, however, have introduced complexity, as the combination of symbols and text can create information overload for drivers [10]. The interpretations of these dashboard indicator lights are explained in the vehicle’s manufacturer’s manual and are required to be easily identified by all drivers irrespective of the language and background. In developing countries like Ghana, a significant proportion of vehicles are imported, with approximately 90% being used vehicles [11,12], some of which are several years old and often without manuals to help drivers interpret these lights. This can lead to confusion and misinterpretation, putting both vehicle and driver safety at risk [3]. Additionally, the prevalence of older, used vehicles could contribute to road safety challenges, as studies have shown that fatalities in older vehicles are four times higher than in newer ones [13].

This study addresses a significant gap in the existing literature by focusing on how drivers understand dashboard indicator lights and how this understanding affects their ability to perform timely maintenance. While indicator lights symbols have received attention, most of these studies focused on the comprehension of the indicator light symbols [14], dashboards and information overload [10], Dashboard layout effects on drivers’ searching performance and heart Rate [2], and effect of different types of indicators to the drive [3]. Additionally, some studies have also explored the technical aspects of these indicators and their role in vehicle diagnostics [9], demonstrating that these indicator lights convey the operational status of vehicle components and safety features. In Ghana only one study examined drivers’ familiarity and comprehension, but it focused on road signs and markings [15]. Although all these studies have contributed significantly to literature, the impact of drivers’ demographic factors and comprehension of indicator light symbols on driver behaviour toward preventive maintenance compliance remain unexamined. Therefore, this study assesses drivers’ familiarity and comprehension of dashboard indicator light symbols and further examines the impact of driver background factors and comprehension level of the symbols on drivers’ preventive maintenance compliance (ensuring proactive vehicle maintenance to prevent breakdowns).

Providing empirical evidence on how different dashboard lights affect driver maintenance behaviour, can inform the design of more intuitive and effective warning systems. For example, many drivers may delay maintenance due to a lack of understanding, leading to potential vehicle damage. Insights from this study can also inform education initiatives aimed at improving driver awareness, leading to better vehicle longevity and performance. Moreso, findings of this study could have significant implications for automotive industries to develop more user-friendly dashboards, and inform policy makers in developing regulations mandating the inclusion of specific warning systems in vehicles. Additionally, while the study focuses on Ghana, the findings may be applicable to regions with similar markets for used vehicles and limited access to vehicle manuals or where driver education on dashboard indicators is insufficient. This can lead to a broader adoption of safety features and improved vehicle maintenance practices among drivers.

## 2. Methodology

### 2.1. Participants

Ethical clearance for this study was obtained from the Research Ethics Committee at the Directorate of Research and Innovation, Ho Technical University, Ghana (HTU/DRI/EC/VOL.I/24/022) to conduct the study. The study objective was explained to participants, and their consent was obtained verbally to voluntarily participate in study under the witness of their colleagues as the participation of individuals in the research poses no risk of physical, psychological, legal, or social harm. The recruitment of the participants commenced on 15^th^ February 2024 and ended on 29^th^ March 2024. All methods of the study were carried out in accordance with relevant guidelines outlined in research ethics policy.

The study was carried out among drivers across the sixteen (16) regions of Ghana. Participants were purposely selected based on their driving experience, as the focus of the study is on individuals who actively interact with dashboard indicator lights. Since these lights provide information and guidance specifically to drivers, individuals without driving experience would not be able to provide appropriate information. Therefore, purposive sampling was employed to ensure that only experienced drivers, who regularly encounter dashboard indicators were included in the study.

Two thousand five hundred and thirty (2530) drivers participated in the study. The participants consist of (i) private vehicle drivers, (ii) commercial vehicle drivers and (iii) both private and commercial vehicle drivers. The study shows that 79% of the participants were males while 21% females as shown in Table 1. Majority of the participants (35.8%) were in the 36–45 years age bracket and had their education up to primary/middle/ junior high school (63.8%). This clearly shows that most of the participants qualify to drive since they are within the required minimum age of 18 years. This is the required age that qualifies a Ghanaian citizen to drive. In addition, most of the participants (57.9%) have a Ghanaian driving licence class B which qualifies them to drive light vehicles, cross country vehicles, etc. Most participants (70.1%) were private car drivers only and (32.9%) had 5–10 years driving experience and drove between 1–4 hours daily (60.5%).

**Table 1:** Demographic characteristics of participants.

Demographic Characteristics	Percentage
***Gender***	
Male	79.0
Female	21.0
***Age (Years)***	
18 - 25	11.8
26 - 35	17.9
36 - 45	35.8
46 - 55	25.4
56 above	9.1
***Educational Level***	
Primary/Middle/ Junior High school	63.8
Secondary school	14.1
Tertiary	18.3
Informal	3.8
***Driving License Category***	
B	57.9
C	21.1
D	13.5
E	3.2
F	4.3
***Vehicle’s often Driven***	
Private car only	70.1
Commercial car only	7.6
Both private and commercial	22.3
***Driving Experience (Years)***	
< 1	2.7
1- 4	25.4
5 - 10	32.9
11-14	18.4
15- 20	10.3
Above 20	10.3
***Daily Hour of Driving***	
1 - 4	60.5
5 -10	25.0
10 -14	14.5

### 2.2. Data collection instrument

The study employed a self-reported questionnaire as the primary data collection instrument. A pilot study was conducted, involving 80 private and commercial drivers to test the clarity and relevance of the questions. Feedback from the pilot participants highlighted the need for rewording to ensure better comprehension and appropriate response. Based on the feedback, adjustments were made to the final survey instrument by rewording the instructions for the response variable (driver compliance to maintenance practices) and suggested improvements to the Likert-scale response options of the question.

The final instrument was divided into two (2) sections. The first section focused on collecting demographic information as shown in Table 1. Besides, the section included a question assessing the extent of action taken by the participants to comply with preventive maintenance practices when the dashboard indicator light symbols illuminate. This response variable was measured using 3-point Likert-scale, ranging from 1 representing ‘No action taken’, 2 denoting ‘Some action taken’, and 3 representing ‘Prompt action taken’. In the second section, participants were presented with 25 coloured images of dashboard indicator light symbols to solicit information on correct identification and understanding of the indicator light symbols. The participants were asked to identify the symbols (see S1 Appendix in the online supporting information) and write down the meaning of each symbol in their own words to assess their understanding levels. Additionally, participants were asked to indicate the appropriateness of indicator light symbols and measured on 3-point Likert-scale, ranging from 1 representing ‘Not appropriate’, 2 denoting ‘Appropriate’, and 3 representing ‘Most appropriate’. The instrument was administered to participants through a face-to-face approach.

Out of the 25 selected indicator light symbols, 10 were classified as warning lights, while engine and emission light and safety light consisted of 5 symbols each. Drivetrain light included 3 symbols and lighting and comfort light each consisted of 1 symbol. These symbols were selected because they are common on vehicles used in Ghana. Table 2 provides a detailed overview of the various indicator light symbols presented to the participant for identification and interpretation. The questions related to these symbols were open-ended to allow for an assessment of participants’ comprehension. After receiving the responses, we categorised the responses and coded as 1 = don’t know, 2 = wrong response, and 3 = correct response. The responses coded 1 were based on the responses where participants explicitly stated that they did not know the meaning of the symbol or failed to provide any response. This Categorization distinguishes between a complete lack of recognition and misinterpretation. It also allowed for an accurate measure of the proportion of participants who had no familiarity with the symbols. The responses coded 2 (wrong response) indicate that the participant attempted to interpret the symbol but provided an incorrect meaning. This category reflects cases where the symbol was recognized but misunderstood. Responses coded 3 (correct responses) indicate that the participant accurately understood and explained the meaning of the symbol as intended. This category reflects a successful comprehension of the indicator lights. This classification method was adapted from the five SAE and ISO categorizations, although categories such as “Partially correct” and “No response given” were excluded due to a lack of responses in these areas [16,17]. The common method bias (potential distortion that may arise in the findings) was controlled by collecting responses from a diverse and representative sample, including private drivers from various workplaces and commercial drivers from different terminals. The approaches encompassed various demographics, experience levels, and license type to minimize selection bias. Additionally, the survey instrument underwent review by experts in human factors, automotive engineering, and traffic psychology to refine questions, eliminate ambiguity, and ensure neutrality, thereby reducing measurement bias. Furthermore, response bias was mitigated by assuring participants anonymity which resolved the assessment worry [18]. Incorporating these measures, enhances the validity and ensures that conclusions drawn are reliable. A total of two thousand five hundred and thirty (2530) participants fully responded to the symbols presented.

**Table 2:** Interpretation of dashboard indicator light symbols.

Name of Panel Indicator Symbol	Meaning	Interpretation (Percentage)		
		Correct	Wrong	Don’t Know
Traction Control/ESP Light	Indicates that there is an issue with the traction control system.	33.4	60.1	6.5
Battery Charge Warning	Indicates that the alternator is not charging the battery properly	89.7	9.7	0.6
Brake Warning Light	Indicates an issue with the brake system and should be checked immediately	66.5	25.8	7.7
Check Engine Light	Indicates an issue with the engine or the emissions	63.8	33.0	3.2
Automatic Shift Lock	Indicates the brake pedal is not pressed out	20.3	70.4	9.3
Low Fuel Level	Indicates low fuel level in the fuel tank or an issue with the fuel level measuring system	50.4	49.6	0.0
Tire Pressure Warning Light	Indicates low air pressure in one or more tires. Indicates that tyre pressure is too low	47.0	53.0	0.0
Airbag Indicator	Indicates a problem in the system and the airbag cannot be inflated in the event of a collision	65.0	35.0	0.0
Coolant Temperature Warning Light	Indicates that the engine coolant temperature is high	54.4	45.6	0.0
Anti-lock Braking System	Indicates there is an issue with your anti-lock brakes or Anti-lock braking system (ABS)	45.7	54.3	0.0
Engine Oil Pressure Light	Indicates low oil pressure in the engine	51.3	48.7	0.0
Glow Plug Indicator (Diesel)	Indicates there is an issue with the glow plugs or the glow plug control system	49.0	51.0	0.0
Hazard Light	Indicates the hazard lights are turned on	48.8	51.2	0.0
Rear Fog Light On	Indicates that the rear fog light lights are turned on	23.3	64.9	11.8
Windshield Defrost Indicator Light	Indicates that the windshield defroster is turned on	60.8	34.4	4.8
Seatbelt Indicator	Indicates one or more seat belts are not closed or there is an issue with the system.	96.1	3.9	0.0
Service Engine Soon	Indicates minor trouble with the engine or low fluid levels	49.6	45.4	4.9
Immobiliser Indicator	Indicates the car key cannot be recognized by the car or there is an issue with the immobiliser system.	41.0	53.0	6.0
4*4 Gear	Indicates that the 4*4 is activated	28.5	65.5	6.0
Washer Fluid Warning Light	Indicate that windshield washer fluid is low	68.5	30.4	1.1
Automatic Transmission Fluid Temperature Light	Automatic Transmission Fluid temperature has increased	43.3	52.3	4.4
Security Door Lock	Indicates a security and anti-theft system has been activated	41.8	54.2	4.0
Power Steering Warning Light	Indicates that there is an issue with the power steering system	35.6	59.5	4.9
Automatic Parking Light	Indicates emergency (parking) brake is engaged	17.7	75.8	6.5
Handbrake Engaged	Indicates handbrake is engaged	34.4	65.6	0.0

### 2.3. Data analysis

The data collected were entered in excel and imported into STATA version 15 for analysis. Descriptive statistics were computed to assess the frequency pattern of the symbols and appropriateness ranking technique used to determine the suitability of the various symbols. Cross tabulation was employed to assess the relationship between familiarity as well as comprehension of the symbols and Chi-square test further conducted to examine the existence of significant differences between the familiarity and comprehension levels of the symbols. Finally, generalised ordered logistic regression analysis was explored to assess the impact of demographic factors and comprehensibility of the indicator light symbols on drivers’ preventive maintenance compliance.

### 2.4. Generalized ordered logistic regression

Generalised Ordered Logistic Regression (GOLR) is a statistical model used when the response variable has more than two ordered categories. This model is an extension of the traditional ordered logistic regression model. In the current study, one of the objectives is to evaluate the impact of demographic factors and comprehension of the indicator light symbols on drivers’ preventive maintenance compliance. The response variable, which is the preventive maintenance compliance, has ordered categories such as ‘No action taken’. ‘Some action taken’, and ‘Prompt action taken’. The predictors include both demographic factors and comprehension of dashboard indicator symbols. The GOLR model estimates the probability of observing each category of the ordinal outcome. The cumulative probabilities for the ordered categories can be modelled using eq. 1:


$$P(Y\leq k|X)=\frac{e^{\beta_{0}^{k}+\beta_{1}^{k}X_{1}+\beta_{2}^{k}X_{2}+\ldots +\beta_{p}^{k}X_{p}}}{1+e^{\beta_{0}^{k}+\beta_{1}^{k}X_{1}+\beta_{2}^{k}X_{2}+\ldots +\beta_{p}^{k}X_{p}}}$$ (1)

Let Y be an ordinal response variable with K ordered categories, and let X be a vector of predictor variables. k=1,2,…,K−1, $\beta_{0}^{k}$ is the threshold parameter for the $k^{th}$ category and $\beta_{j}^{k}$ are the coefficients associated with the predictor variables for the $k^{th}$ category.

To define the cumulative odds for each category, the model is express as indicated in eq. 2:


$$log\left(\frac{P(Y\leq k|X)}{P(Y>k|X)}\right)=\beta_{0}^{k}+\sum_{j=1}^{p}\;\beta_{j}^{k}X_{j}$$ (2)

The parameters were estimated using maximum likelihood estimation (MLE). The likelihood function was constructed based on the observed outcomes and was maximised to obtain the estimates of the parameters β. $\beta_{0}^{k}\;$ represent the threshold between the categories of the outcome variable. $\beta_{j}^{k}\;$ is for each coefficient indicating the change in the log odds of being in a category k or lower versus being in a higher category for a one unit increase in the predictor variable.

## 3. Results and discussion

### 3.1. Monitoring and familiarity of indicator light symbols

**Fig 1:**
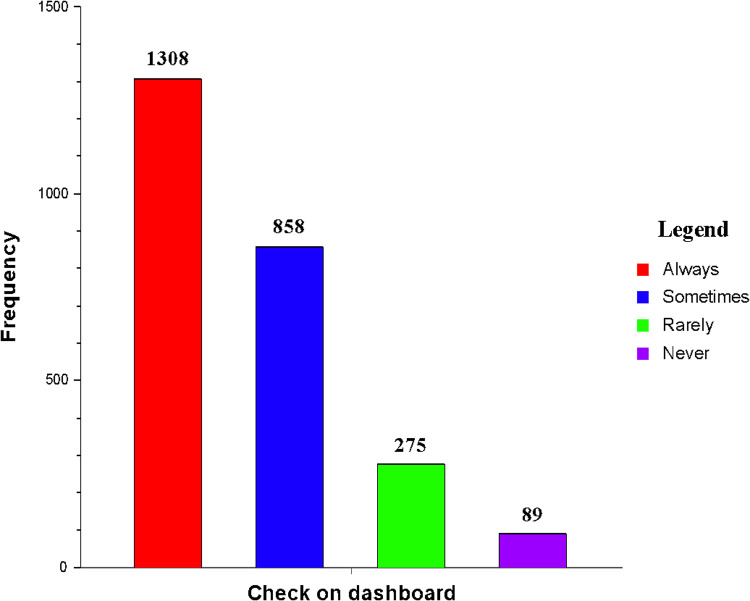
Rates of monitoring panel indicator light symbols.

According to the results, the majority of the participants 1308 (51.7%) always looked at their instrument cluster when driving (see Fig 1). This is one of the best practices and principles when driving. This serves as an alert guide to drivers, providing warnings and directives on precautionary measures to prevent accidents and enhance vehicle safety [19].

**Fig 2:**
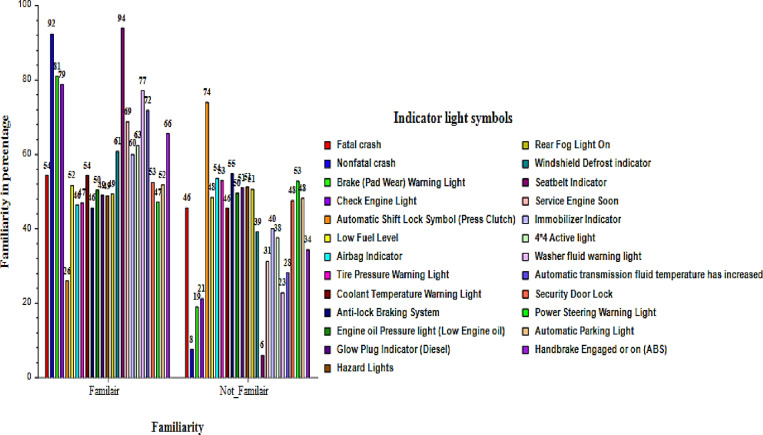
Familiarity of the dashboard indicator light symbols.

Generally, the result in Fig 2 indicates that a higher percentage of participants were familiar with most of the dashboard indicator light symbols. Out of the 25 symbols presented, most participants recognized 16 (64%) of the symbols. Seat Belt indicator symbols have the highest percentage of familiarity (94.0%). This is not surprising because all vehicles are equipped with seatbelts which is one of the passive safety devices. The seatbelts are worn to minimise damages and reduce the risk of injuries to vehicle occupants (i.e., driver and passengers) during the time of impact or crash [20]. The battery charging warning indicator symbol light was the second highest to be recognized (92%). This could be attributed to the fact that the battery is the main source of power for the vehicle with any associated fault being signalled onto the dashboard for the driver to detect. Most of the drivers might have witnessed any battery related challenge accounting for the second highest identification and recognition. On the contrary, the automatic shift lock symbol received the least recognition (26%) by participants. This could be due to the fact that it is only associated with automatic transmitted vehicles which some of participants might have not used.

### 3.2. Interpretation of dashboard indicator light symbols

Generally, out of the 25-dashboard indicator light symbols presented, participants interpreted 11 (44%) correctly and 14 (56%) wrongly. Seatbelt indicator symbol received the highest correct interpretation of 96.1% which could be attributed to it being common in all vehicles and related to safety of vehicle’s occupants. Additionally, it could also be that the majority of vehicles now used in Ghana have visual and auditory alerts for all seats. This was followed by battery charging warning light symbol (89.7%) which might also be due battery being the main power source of all vehicles and thus a very important feature drivers must know about. On the contrary, the automatic parking light and automatic shift lock dashboard indicator light symbols received (75.8%) and (70.4%) wrong responses respectively. This could probably be due to the association of these symbols to automatic transmitted vehicles only, which some of the participants might have not driven. In general, the percentages of dashboard indicator light symbols that the participants don’t know were very low with rear fog light symbols recording 11.8%, which is the highest percentage. This is because most vehicles used do not have rear fog lights, leading to lower understanding among participants.

The International Standards Organization (ISO) recommends that 85% correct response of graphical symbols indicate a well-known symbol [21,22]. Using the ISO criterion, dashboard indicator symbol lights with correct responses of 85% and above were regarded as having high understanding and well recognized, whereas correct responses with less than 85% were classified as having low understanding and poorly recognized. Out of the 25 symbols, only two symbols namely battery charge warning and seatbelt indicator symbols had correct responses greater than 85%; an indication of high understanding and well interpretation of the symbols. This is not surprising since these two dashboard indicator symbols have pictorial design and are very important and common in all vehicles. Thus, high understanding and interpretation are regarded as familiarity of the symbol which implies a complete state of knowing the symbols very well.

### 3.3. Comprehensibility of dashboard indicator light symbols

Table 3 was used to categorise drivers’ comprehensibility into nine comprehension scores for easy comparison among the symbols. This nine-score comprehension categorization was adopted from the study of Campbell et al. [14], which conducted comprehension testing of active safety symbols. As shown in Table 4, the categorization ranges from intended exact matching of the symbols to the meaning (1) to critical confusions or errors in the meaning leading to unsafe action (9).

**Table 3:** Rating scales for categorising and scoring comprehensibility.

Comprehension Score	Description
1	The response matches the intended meaning of the icon exactly.
2	The response captures all major informational elements of the intended meaning of the icon, but is missing one or more minor informational elements.
3	The response captures some of the intended meaning of the icon, but it is missing one or more major informational elements.
4	The response does not match the intended meaning of the icon, but it captures some major or minor informational elements.
5	The response does not match the intended meaning of the icon, but it is somewhat relevant.
6	Participant’s response is in no way relevant to the intended meaning of the icon.
7	Participants indicated he/she did not understand the icon.
8	No answer.
9	For safety-critical icons, identify the number and percentage of critical confusions or errors. Critical confusions or errors reflect responses that indicate that the subject perceived the message to convey a potentially unsafe action.

**Table 4:** Dashboard indicator light symbols comprehension ratings in percentage.

Name of Panel Indicator Symbol	1	2	3	4	5	6	7	8	9	1-2 High	3-4 Low	5-8 None	9 Critic Con
Traction Control/ESP Light	13.3	24.6	10.5	0.0	0.0	7.6	13.5	30.5	0.0	37.9%	10.5%	51.6%	0.0%
Battery Charge Warning	19.0	16.2	42.9	5.7	1.0	1.0	1.0	13.2	0.0	35.2%	48.6%	16.2%	0.0%
Brake Warning Light	2.9	16.2	4.8	21.0	0.0	17.1	11.4	26.6	0.0	19.1%	25.8%	55.1%	0.0%
Check Engine Light	45.7	2.9	1.0	2.9	0.0	14.3	5.7	27.5	0.0	48.6%	3.9%	47.5%	0.0%
Automatic Shift Lock	1.9	2.9	9.5	0.0	0.0	8.6	19.0	58.1	0.0	4.8%	9.5%	85.7%	0.0%
Low Fuel Level	82.9	5.7	0.0	0.0	0.0	1.0	0.0	10.4	0.0	88.6%	0.0%	11.4%	0.0%
Tire Pressure Warning Light	41	2.8	0.0	0.0	0.0	6.7	11.5	38	0.0	41.0%	2.8%	56.2%	0.0%
Airbag Indicator	42.9	0.0	0.0	0.0	0.0	39	1.0	17.1	0.0	42.9%	0.0%	57.1%	0.0%
Coolant Temperature Warning Light	61.8	0.0	0.0	0.0	0.0	8.7	9.5	20.0	0.0	61.8%	0.0%	38.2%	0.0%
Anti-lock Braking System	27.6	19.0	5.7	1.0	0.0	13.3	10.5	22.9	0.0	46.6%	6.7%	46.7%	0.0%
Engine Oil Pressure Light	66.6	0.0	0.0	0.0	0.0	6.7	4.8	21.9	0.0	66.6%	0.0	33.4%	0.0%
Glow Plug Indicator (Diesel)	18.1	2.9	1.8	0.0	0.0	4.8	20.0	52.4	0.0	21%	1.8%	77.2%	0.0%
Hazard Light	36.2	1.9	1.9	1.0	0.0	8.6	11.4	39.0	0.0	38.1%	2.9%	59%	0.0%
Rear Fog Light On	15.2	1.0	0.0	21.0	1.0	4.8	11.3	45.7	0.0	16.2%	21.0%	62.8%	0.0%
Windshield Defrost Indicator Light	10.5	7.6	3.8	6.7	0.0	24.8	9.5	37.1	0.0	18.1%	10.5%	71.4%	0.0%
Seatbelt Indicator	53.3	1.0	0.0	0.0	0.0	1.9	4.8	39.0	0.0	54.3%	0.0%	45.7%	0.0%
Service Engine Soon	45.7	0.0	0.0	0.0	0.0	3.8	7.6	42.9	0.0	45.7%	0.0%	54.3%	0.0%
Immobiliser Indicator	9.5	19.0	2.9	0.0	0.0	2.9	12.4	53.3	0.0	28.5%	2.9%	68.6%	0.0%
4*4 Gear	3.8	4.8	3.7	0.0	1.0	1.0	17.1	68.6	0.0	8.6%	3.7%	87.7%	0.0%
Washer Fluid Warning Light	63.8	0.0	0.0	0.0	0.0	1.0	3.8	31.4	0.0	63.8%	0.0%	36.2%	0.0%
Automatic Transmission Fluid Temperature Light	19.0	3.8	0.0	0.0	0.0	16.2	6.7	54.3	0.0	22.8%	0.0%	77.2%	0.0%
Security Door Lock	1.9	3.8	7.6	2.8	0.0	1.0	14.3	68.6	0.0	5.7%	10.4%	83.9%	0.0%
Power Steering Warning Light	11.3	14.3	1.9	1.0	0.0	1.0	10.5	60.0	0.0	25.6%	2.9%	71.5%	0.0%
Automatic Parking Light	1.0	0.0	0.0	0.0	0.0	9.5	17.1	72.4	0.0	1.0%	0.0%	99.0%	0.0%
Handbrake Engaged	18.1	24.8	11.4	1.0	0.0	6.7	1.9	36.2	0.0	42.9%	12.4%	44.7%	0.0%

The categorisation provides a structured framework for assessing how accurately participants interpret symbols, ranging from precise understanding to misinterpretation or lack of response. This categorization systematically evaluates comprehension levels, distinguishing varying degrees of accuracy and relevance in participants’ interpretations. Using this rating scale, identified areas for improving symbol clarity and effectiveness.

Based on the categorization ratings as indicated in Table 4, the first column labelled “1-2 High,” is defined as high comprehension which results from the combination of 1 and 2 scoring categories. The column labelled “3-4 Low” is defined as low comprehension was formed from the combination of scores from scoring categories 3 and 4. The column labelled “5-8 None” is defined as no comprehension and a reflection of combined scores from scoring categories 5,6, 7 and 8. The column labelled “9 Crit. Con.” Is defined as critical confusion which is scores from scoring category 9. The critical confusions or errors category represent responses by participants that do not match the exact meaning. For instance, a forward collision warning symbol being perceived by a participant to indicate a rear collision [14].

It can be observed that out of the 25 symbols presented, 6 (24%) had high comprehension, 1 (4%) were comprehended low and majority 18 (72%) had no comprehension across board. The results clearly show that participants were not able to comprehend most of the dashboard indicator light symbols. The six highly comprehended symbols were “check engine light”, “low fuel level”, “coolant temperature warning light”, “engine oil pressure light”, “seatbelt indicator” and “airbag indicator symbol lights’‘. These highly comprehended indicator light symbols are commonly seen on vehicle dashboards and drivers are likely to recognize and comprehend appropriately. Generally, none of the symbols was associated with the critical confusion category of rating. These symbols are effectively communicated to drivers and are likely to be recognized and responded to appropriately in various driving situations.

### 3.4. Appropriateness of the dashboard indicator light symbols

The appropriateness ranking technique [23] was employed to determine the suitability of the various symbols. Table 5 shows the appropriateness ranking of the dashboard indicator light symbols. The symbols were ranked into three categories namely ‘most appropriate’, ‘appropriate’ and ‘not appropriate’. It was observed that low fuel level symbol was ranked as the most appropriate with 82.9%, followed by engine oil pressure light symbol (66.6%) and seatbelt indicator symbol (53.3%). This could be due to the familiarity of these symbols since they are common and related to all vehicles. In addition, they are warning indicator lights which call for much concern. However, automatic parking light and 4*4 engagement gear symbols were ranked not appropriate with 99% and 87.7% respectively. In addition, the security door lock symbol was the third ranked as not appropriate with 86.7%. These dashboard indicator symbols are associated with some specific vehicles and not common to all vehicles which might account for their unfamiliarity to most drivers.

**Table 5:** Appropriateness ranking of dashboard indicator light symbols.

Name of Panel Indicator Symbol	Most Appropriate (%)	Appropriate (%)	Not Appropriate (%)
Traction Control/ESP Light	13.3	35.1	51.6
Battery Charge Warning	19.0	59.1	21.9
Brake Warning Light	2.9	21.0	76.10
Check Engine Light	45.7	3.9	50.4
Automatic Shift Lock	1.9	12.4	85.7
Low Fuel Level	82.9	5.7	11.40
Airbag Indicator	42.9	0.0	57.10
Tire Pressure Warning Light	41.0	2.8	56.2
Coolant Temperature Warning Light	61.8	0.0	38.2
Anti-lock Braking System	27.6	24.7	47.7
Engine Oil Pressure Light	66.6	0.0	33.4
Glow Plug Indicator (Diesel)	18.1	4.7	77.2
Hazard Light	36.2	3.8	60.0
Rear Fog Light On	15.2	1.0	83.8
Windshield Defrost Indicator Light	10.5	11.4	78.1
Seatbelt Indicator	53.3	1.0	45.7
Service Engine Soon	45.7	0.0	54.3
Immobiliser Indicator	9.5	21.9	68.6
4*4 Gear	3.8	8.5	87.7
Washer Fluid Warning Light	63.80	0	36.2
Automatic Transmission Fluid Temperature Light	19.00	3.8	77.2
Security Door lock	1.9	11.4	86.7
Power Steering Warning Light	11.3	16.2	72.5
Automatic Parking Light	1.0	0.0	99.0
Handbrake Engaged	18.1	36.2	45.8

Across board, the appropriateness ranking analysis was very consistent with the comprehensibility assessment on low fuel level, engine oil pressure light and seatbelt, coolant temperature and washer fluid warning light indicator symbols being highly comprehended. This result was in agreement with a similar study that evaluated in-vehicle symbols for an intersection avoidance system [24] and powertrain and Advanced Driver Assistance Systems [16]. This suggests that drivers tend to accurately recognize and interpret dashboard symbols that are critical to vehicle operation and safety. Possibly, these indicators are well-designed and possibly reinforced through driver experience and training.

### 3.5. Cross-tabulation of familiarity and comprehensibility of the dashboard indicator light symbols

Even though the prior result has shown that the percentage of symbol familiarity were greater than the comprehension, it is possible that drivers may be very familiar with the dashboard indicator light symbols, but may not/or may comprehend the symbols. Hence, comprehension level (i.e., whether drivers understand the message or information communicated by the symbols) of drivers who are familiar (i.e., the state of knowing the symbols very well) with the symbols was determined. The existence of significant differences between the familiarity and comprehension levels of the symbols was further examined using a Chi-square test. The Chi-square analysis was based on the following null and alternative hypothesis that:

H0: The familiarity and comprehension level of dashboard indicator light symbols are independent of each other (null hypothesis).

H1: The familiarity and comprehension level of dashboard indicator light symbols are dependent on each other (alternative hypothesis).

From Table 6, it was observed that drivers who were familiar with dashboard indicator light symbols, the majority comprehended them. This finding corresponds with previous studies on road traffic signs [13]. These could be attributed to the fact that these symbols are very common and might often be observed by most drivers. Likewise, the proportion of drivers who were not familiar with the symbols, majority did not comprehend the symbols. This finding implies that these categories of drivers may have been ignoring the warning signs and they are likely not motivated to check vehicle status when these lights signal on the vehicle dashboard.

**Table 6:** Cross-tabulation analysis of familiarity and comprehension level.

Name of Panel Indicator Symbol	Meaning	Familiarity	No of Participants (Figures in bracket are %)	Comprehension level (%)		Chi-square	Sig. (2-sided)	H_0_ Rejected?
				Comprehended	Not Comprehended			
Traction Control/ESP Light	Indicates that there is an issue with the traction control system.	Familiar	1374 (54.3)	59.9	40.1	843.18	0.000	YES
		Unfamiliar	1156 (45.7)	2.5	97.5			
Battery Charge Warning	Indicates that the generator (or alternator) is not charging the battery properly	Familiar	2338 (92.4)	93.5	6.5	318.65	0.000	YES
		Unfamiliar	187 (7.6)	61.5	38.5			
Brake Warning Light	Indicates an issue with the brake system and should be checked immediately	Familiar	2049 (81)	80.0	20.0	687.934	0.000	YES
		Unfamiliar	481 (19)	11.0	89.0			
Check Engine Light	Indicates an issue with the engine or the emissions	Familiar	1994 (78.8)	83.4	16.6	1121.987	0.000	YES
		Unfamiliar	536 (21.2)	5	95			
Automatic Shift Lock	Indicates the brake pedal is not pressed out	Familiar	658 (26)	80.0	20.0	1492.64	0.000	YES
		Unfamiliar	1872 (74)	0.8	99.2			
Low Fuel Level	Indicates low fuel level in the fuel tank or an issue with the fuel level measuring system	Familiar	1308 (51.7)	97.4	2.6	2115.11	0.000	YES
		Unfamiliar	1222 (48.3)	3	97			
Tire Pressure Warning Light	Indicates low air pressure in one or more tyres	Familiar	1189 (47.0)	94	6	2232.000	0.00	YES
		Unfamiliar	1341 (53.0)	2.8	97.2			
Airbag Indicator	Indicates a problem in the system and the airbag cannot be inflated in the event of a collision	Familiar	1171 (46.3)	98.5	1.5	2172.42	0.000	YES
		Unfamiliar	1359 (53.7)	5.4	94.6			
Coolant Temperature Warning Light	Indicates that the engine coolant temperature is high	Familiar	1376 (54.4)	92	8	2122.000	0.000	YES
		Unfamiliar	1154 (45.6)	1.2	98.8			
Anti-lock Braking System	Indicates there is an issue with your anti-lock brakes or Anti-lock braking system (ABS)	Familiar	1156 (45.7)	93.3	6.7	2186.000	0.000	YES
		Unfamiliar	1374 (54.3)	2.9	97.1			
Engine Oil Pressure Light	Indicates low oil pressure in the engine	Familiar	1275 (50.4)	100	0	2162.690	0.000	YES
		Unfamiliar	1255 (49.6)	1.3	98.7			
Glow Plug Indicator (Diesel)	Indicates there is an issue with the glow plugs or the glow plug control system	Familiar	1240 (49.0)	96.4	3.6	1231.63	0.000	YES
		Unfamiliar	1290 (51.0)	0	100			
Hazard Light	Indicates the hazard lights are turned on	Familiar	1235 (48.8)	100	0	1423.298	0.000	NO
		Unfamiliar	1295 (51.2)	0	100			
Rear Fog Light On	Indicates that the rear fog light lights are turned on	Familiar	1250 (49.4)	37.9	72.1	369.604	0.000	YES
		Unfamiliar	1280 (50.6)	7.7	92.3			
Windshield Defrost Indicator Light	Indicates that the windshield defroster is turned on	Familiar	1538 (60.8)	81.0	19	437.31	0.000	YES
		Unfamiliar	992 (39.2)	35.2	64.8			
Seatbelt Indicator	Indicates one or more seat belts are not closed or there is an issue with the system. Check both the driver and passenger sides and the rear seats on some car models.	Familiar	2378 (94.0)	98.1	1.9	135.725	0.000	YES
		Unfamiliar	152 (6.0)	80.0	20			
Service Engine Soon	Indicates minor trouble with the engine or low fluid levels	Familiar	1738 (68.7)	63.8	36.2	557.995	0.00	YES
		Unfamiliar	792 (31.3)	12.9	87.1			
Immobiliser Indicator	Indicates the car key cannot be recognized by the car or there is an issue with the immobiliser system.	Familiar	1521 (60.1)	64.4	35.6	808.380	0.00	YES
		Unfamiliar	1009 (39.9)	5.7	94.3			
4*4 Gear	Indicates that the 4*4 is activated	Familiar	1581 (62.5)	45.6	54.4	524.92	0.000	YES
		Unfamiliar	949 (37.5)	1.6	98.4			
Washer Fluid Warning Light	Indicate that windshield washer fluid is low	Familiar	1953 (77.2)	29.4	70.6	202.576	0.000	YES
		Unfamiliar	577 (22.8)	6.9	93.1			
Automatic Transmission Fluid Temperature Light	Automatic Transmission Fluid temperature has increased	Familiar	1817 (71.8)	59.7	40.3	530.700	0.000	YES
		Unfamiliar	713 (28.2)	6.3	93.7			
Security Door Lock	Indicates a security and anti-theft system has been activated	Familiar	1328 (52.5)	69.7	30.3	1441.057	0.000	YES
		Unfamiliar	1202 (47.5)	9.5	90.5			
Power Steering Warning Light	Indicates that there is an issue with the power steering system	Familiar	1194 (47.2)	65.8	43.2	767.794	0.000	YES
		Unfamiliar	1336 (52.8)	9.0	91.0			
Automatic Parking Light	Indicates emergency (parking) brake is engaged	Familiar	1311 (51.8)	36.0	64	466.024	0.000	YES
		Unfamiliar	1219 (48.2)	12	88			
Handbrake Engaged	Indicates handbrake is engaged	Familiar	1660 (65.6)	51.4	48.6	437.254	0.013	YES
		Unfamiliar	870 (34.4)	7.1	92.9			

However, it is interesting to know that even though, drivers were familiar with some symbols like Rear fog light”, “4x4 gear”, “washer” and “Automatic parking light”, the majority (72%, 54.4%, 70.6% and 64% respectively) did not comprehend the meaning of these symbols. This suggests that drivers could recognize the indicator light symbols, they are likely to comprehend wrongly. In line with study on road signs the finding implies that familiarity with dashboard indicator light symbols does not necessarily mean that drivers understand [25].

Similarity, it was thought-provoking to note that although some drivers were unfamiliar with symbols such as “Battery lights warning” and “Seat belts warning” on their dashboard, the majority (61% and 80% respectively) of these drivers were able to understand the meanings conveyed by these symbols. It could be that because red lights signify urgent action, there is a level of common knowledge among drivers regarding these dashboard warning signs, even if they are not familiar with these symbols.

Taking into consideration the 5% level of significance and the critical chi-square value of 3.84, it was observed that the estimated chi-square values for all the dashboard indicator lights were higher than the critical chi-square value indicating that there is a statistical difference between familiarity and comprehension. This explains that familiarity and comprehension of these indicator symbols are significantly associated and dependent on each other. Hence, the null hypothesis is rejected. This confirmed that drivers who were familiar with these indicator light symbols were more likely to comprehend than those who were not familiar. The result supports the findings of previous studies that familiarity will improve comprehension [15]. This confirmed that drivers who were familiar with these indicator light symbols were more likely to comprehend than those who were not familiar.

### 3.6. Generalized ordered logistic regression model

The demographic factors such as educational background, class of driving licence, years of driving experience and attitude of regularly checking the instruments panel as well as comprehensibility of various dashboard indicator light symbols were entered as independent variables in the model. Driver’s preventive maintenance compliance was used as a response variable. This indicates whether drivers take action, such as checking their vehicle’s status or addressing any issues, when they see the indicator lights illuminate on the vehicle dashboard. Action might involve inspecting the light, or seeking assistance from a mechanic. In this study, a p-value of 0.05 suggests that there is enough evidence to conclude that there is a significant relationship observed.

#### 3.6.1. Impact of demographic factors on preventive maintenance compliance.

The result in Table 7 shows that a driver with secondary and tertiary education background has a positive significant association with drivers’ preventive maintenance compliance with an odd ratio of 2.297 and 4.414 respectively, which suggests that for every one unit increase in secondary and tertiary educational background, the odds of drivers’ preventive maintenance compliance are 2.3 and 4.4 times higher respectively when the dashboard indicator light appears. This indicates that drivers with higher levels of education are more likely to initiate preventive maintenance of their vehicles, especially when they observe an indicator light appearing on the dashboard. In accordance with previous study, this could be attributed to a greater understanding of the importance of vehicle maintenance and safety [3,4] among individuals with higher educational attainment. Similarly, a previous study demonstrated a notable correlation between drivers’ educational backgrounds and their propensity to engage in vehicle maintenance behaviours [26], particularly in response to symbols.

**Table 7:** Estimated model results.

Parameter	B	Std. Error	Sig.	Exp(B)	95% Wald Confidence Interval for Exp(B)	
					Lower	Upper
**Educational background**						
Primary/Middle/ Junior High school	3.744	7.194	0.603	42.265	3.18	561.800
Secondary school	0.832	0.382	0.023	2.297	1.186	2.998
Tertiary	1.485	0.745	0.042	4.414	3.436	5.670
Informal	2.423	7.172	0.736	11.274	8.86	143.220
**Class of License**						
B	-5.936	0.859	0.000	0.003	0.000	0.014
C	-2.258	0.758	0.003	0.105	0.024	0.462
D	1.299	0.322	0.000	3.665	1.001	1.032
E	1.132	0.367	0.001	3.102	1.007	1.261
F	–	–	–	–	–	–
**Driving Experience (Years)**						
< 1	-1.262	0.5160	0.014	0.283	0.103	.778
1- 4	-1.325	1.338	0.322	0.266	0.019	3.664
5 - 10	-1.961	1.347	0.145	0.141	0.010	1.973
11-14	0.865	0.4202	0.040	2.375	1.042	5.412
15- 20	0.648	0.227	0.011	1.912	1.002	1.427
Above 20	–	–	–	–	–	–
**Regular Check on Dashboard**						
Always	1.677	0.309	.000	5.349	2.008	3.083
Sometimes	0.808	0.214	.000	2.243	1.007	2.074
Rarely	0.108	3.979	0.978	1.114	0.576	271.017
Never	–	–	–	–	–	–
**Comprehensibility of Warning Lights Symbols**						
Traction Control/ESP Light	-0.484	0.252	0.051	0.616	0.376	1.011
Battery Charging Warning Light	-4.760	0.510	0.000	0.009	0.003	0.023
Brake Pad Wear Warning Light	0.498	0.206	0.016	1.645	1.099	2.463
Low Fuel Level	0.520	0.254	0.041	1.683	1.022	2.771
Tyre Pressure Warning Light	-0.622	0.294	0.034	0.537	0.302	0.955
Coolant Temperature Warning Light	-0.009	0.266	0.974	0.991	0.588	1.670
Anti-lock Braking System	-0.032	0.223	0.886	0.968	0.625	1.500
Immobiliser Indicator	1.749	0.263	0.000	5.751	3.435	9.630
Power Steering Warning Light	-1.222	0.230	0.000	0.295	0.188	0.463
Hazard Light	0.510	0.323	0.114	1.666	0.885	3.136
Washer Fluid Warning Light	2.398	0.318	0.000	11.001	5.899	20.517
**Comprehensibility of Safety Lights Symbols**						
Seatbelt Indicator	-2.459	0.777	0.002	0.086	0.019	0.393
Airbag Indicator	0.299	0.302	0.323	1.348	0.745	2.438
Automatic Parking Light	1.872	0.355	0.000	6.499	3.243	13.021
Handbrake Engaged or on (ABS)	1.206	0.359	0.001	3.339	1.653	6.747
Security Door Lock	1.504	0.268	0.000	4.500	2.661	7.611
**Comprehensibility of Engine and Emission Lights Symbols**						
Engine due for Servicing Soon	1.927	0.334	0.000	6.868	1.028	2.103
Check Engine Light	1.120	0.343	0.001	3.066	1.565	6.006
Engine Oil Pressure Light	-0.334	0.266	0.210	0.716	0.425	1.206
Glow Plug Indicator (Diesel)	-0.073	0.276	0.792	0.930	0.542	1.597
**Comprehensibility of Drivetrain Light Symbols**						
4*4 Active light	-0.826	0.272	0.002	0.438	0.257	0.745
Automatic Transmission Fluid Temperature	7.590	0.482	0.000	1977.70	769.20	5084.862
Automatic Shift Lock Symbol	-0.155	0.248	0.531	0.856	0.526	1.393
**Comprehensibility of Lights Symbol**						
Rear Fog Light On	-0.275	0.216	0.204	0.760	0.497	1.161
**Comprehensibility of Comfort Lights Symbol**						
Windshield Defrost Indicator	0.950	0.258	0.000	2.586	1.561	4.285

−2 Log L =866.473; LR (Chi-sq) = 1465.216; p-value < 0.001; AIC = 1818.946; BIC = 2060.636.

The result further indicates a negative significant relationship between drivers’ preventive maintenance compliance and drivers with licence B and C, suggesting that drivers with licence B and C are 0.003 and 0.105 times less likely, respectively, to comply with preventive maintenance practices when a dashboard indicator light illuminates. Conversely, holders of licences D and E display a markedly different pattern of action, being significantly more motivated to address maintenance issues promptly. This is evidenced by higher odds ratios of 3.665 and 3.102 respectively. This suggests that drivers with licences D and E are approximately 3.7 and 3.1 times more likely, respectively, to comply with preventive maintenance practices when a dashboard indicator light appears.

Regarding driving experience, drivers with less than 1 year of experience and those with 5–10 years of experience show negative significant coefficients indicating decreased likelihood of driver’s compliance to preventive maintenance practices when the dashboard indicator lights appear. Possibly, novice drivers with less than 1 year of experience may lack the familiarity and confidence to interpret dashboard indicators accurately or may prioritise other aspects of driving over vehicle maintenance. In line with previous study, these drivers may lack the confidence to address maintenance issues independently [3,4]. Similarly, drivers with 5–10 years of experience may have settled into driving habits and routines, potentially becoming complacent to maintenance-related signals.

Conversely, drivers with 11–14 and 15–20 years driving experience are 2.375 and 1.912 times more likely to comply with preventive maintenance when the dashboard indicator lights illuminate. These drivers likely have extensive driving experience and knowledge over the years, including the importance of proactive vehicle maintenance. Perhaps, experienced drivers may have encountered various maintenance issues and appreciate the importance of addressing dashboard indicators promptly to ensure vehicle safety and performance. The finding is contrary to the previous study, which found driving experience as not a contributing factor to correctly identifying a symbols’ meaning nor what action to take in response to the symbol [4].

Similarly, drivers who regularly and sometimes check on the dashboard while driving are 5.349 and 2.243 times respectively more likely to comply with preventive maintenance practices when the dashboard indicator lights illuminate compared to drivers who rarely or never check on the vehicle dashboard. This finding suggests that habitual dashboard monitoring heightened vigilance of the driver to detect and respond to changes in vehicle status promptly. While some drivers may not consistently monitor the dashboard, their occasional habit may still contribute to a greater awareness of vehicle status.

#### 3.6.2. Impact of warning lights symbols on preventive maintenance compliance.

The result in Table 7, further shows that there is a relationship between warning lights and drivers preventive maintenance compliance. Specifically, the results show that the activation of Traction Control/ESP, Battery Charging, Tyre Pressure, and Power Steering warning lights have a negative significant relationship with drivers’ preventive maintenance compliance. The observed result suggests that when these warning lights are activated, drivers are 0.616, 0.009, 0.537and 0.295-times respectively less likely to respond to maintenance issues. This could be due to a variety of factors, such as ignorance of the significance of the warning lights or a perception that the warning lights are not indicative of serious issues. For instance, low understanding of a warning symbol for low tyre pressure may result in driving with underinflated tyres, leading to reduced vehicle control and increased risk of accidents. Therefore, measures to facilitate prompt preventive maintenance when faced with vehicle-related warnings or malfunctions is essential.

On the other hand, Brake Pad Wear, Low fuel level, Immobilizer Indicator and Washer fluid warning light on vehicle dashboard were significantly positive related to drivers’ preventive maintenance compliance with an odd of 1.645, 1.683, 5.751, 11.001 respectively. This means that prompt maintenance compliance of drivers is likely to increase for every one unit increase in the activation of Brake Pad Wear, Low Fuel Level, Immobilizer Indicator, and Washer fluid warning lights on dashboard. Overall, these findings indicate that certain warning lights significantly prompts drivers to take corrective actions to address the issue [27]. Similarly, studies found that symbols related to basic vehicle functions have significantly higher safety scores [3,4]. Overall, these findings imply that certain warning lights significantly motivate drivers to take corrective actions to respond to maintenance issues promptly, due to concerns about safety, functionality, convenience and operational issues related to these lights.

#### 3.6.3. Impact of safety light symbols on preventive maintenance compliance.

The result reveals a significant negative relationship between the comprehensibility of the seat belt indicator light and drivers’ preventive maintenance compliance, with an odds ratio of 0. 086. This finding suggests that for every one-unit increase in the comprehensibility of the seat belt indicator light, the likelihood of drivers complying to preventive maintenance practices decreases by 0.086 times. Consequently, as the comprehensibility of the seat belt indicator light improves, drivers may feel less compelled to take proactive maintenance measures to ensure that this safety feature is operational. This could be attributed to implicitly trusting the clarity of the seat belt indicator light, potentially leading to decreased vigilance regarding vehicle safety. It may also imply a trade-off between the clarity of the seat belt indicator light and the inclination of individuals to respond to the signal.

The findings also indicate significant positive effect of comprehensibility of “Automatic Parking Light”, “Handbrake engagement (ABS)”, and “Security Door Lock” indicators on drivers’ preventive maintenance compliance, with odds ratios of 6.499, 3.339, and 4.500, respectively. This implies that as comprehension of these dashboard indicators increases by one unit, there is a notable increase in the likelihood of drivers promptly complying with maintenance practices to respond to these signals. It could be that a better understanding of these dashboard indicators prompts them to take action to ensure the proper functioning and safety of the vehicle [3,4] thereby contributing to the maintenance of the vehicle, reducing the risk of vehicle breakdown.

#### 3.6.4. Impact of engine and emission lights symbols on preventive maintenance compliance.

The notable positive estimation values for the “Engine due for Servicing Soon” and “Check engine light” as indicated in Table 7 suggests a relatively strong positive relationship between drivers’ comprehension of these dashboard indicators and preventive maintenance compliance with an odd ratio of 6.868 and 3.066 respectively. This means that for every one unit increase in the driver’s comprehension of these dashboard indicators activated, the odds of drivers promptly responding to preventive maintenance practices increase by approximately 6.868 and 3.066 times. Consequently, drivers who understand the significance of the symbol “Engine due for Servicing Soon” are notably more likely to take action to carry out maintenance on their vehicle, such as scheduling a servicing appointment or performing basic checks themselves. Similarly, drivers understanding the implications of the “Check Engine Light” symbol, are more likely to promptly address any maintenance issues with their vehicle. Similar to previous studies [3,4], these findings suggest strong association between comprehension of these symbols and proactive response to vehicle maintenance signals, ultimately contributing to enhanced safety reliability, and longevity of the vehicle.

#### 3.6.5. Impact of drivetrain light symbols on preventive maintenance compliance.

The results indicate a significant negative association between comprehension of the “4x4 Active light” and drivers’ preventive maintenance compliance, with an odds ratio of 0.438. Conversely, comprehension of “Increased Automatic Transmission Fluid Temperature” symbol is positively associated with drivers’ preventive maintenance compliance, with an odds ratio of 1977.1701. This implies that drivers who understand the “4x4 active light” are approximately 0.438 times less likely to take corrective action when the 4x4 system is activated but the indicator light does not appear on the dashboard. In contrast, drivers are 1977.17 times more likely to comply with maintenance practices when they comprehend the “Increased Automatic Transmission Fluid Temperature” symbol. These findings align with previous research, which demonstrates that drivers who understand powertrain indicator lights are significantly more likely to take prompt action to check their vehicle’s status [4], although comprehension of certain drivetrain indicators may decrease the likelihood of taking immediate maintenance actions.

#### 5.6.6. Impact lights symbol on preventive maintenance compliance.

It was observed that comprehension of the “Rear fog light” indicator is not significantly related to driver’s preventive maintenance compliance (p-value = 0.204), with an odd ratio of 0.760. The observed result suggests that when the “Rear fog light” indicator fails to illuminate on the dashboard when activated, it is unlikely to influence drivers’ [28] to take action to comply with maintenance practices. When the indicator is on even when the rear fog lights are off, it could be a sign of a burned-out bulb or issue with the vehicle’s electrical system (e.g., faulty wiring or a malfunctioning switch). This would require attention to prevent further electrical issues. Perhaps, the lack of significant relationship could be that drivers either do not perceive the rear fog light indicator as important or misunderstand its significance.

#### 3.6.7. Impact comfort lights symbol on preventive maintenance compliance.

The result indicates a significant positive association between the comprehension of the “Windshield Defrost indicator” and driver’s preventive maintenance compliance with an odd ratio of 2.586. This finding suggests that drivers are approximately 2.6 times more likely to take corrective action when the defroster is activated, but the corresponding indicator does not appear on the dashboard. This implies that drivers understand the significance of the “Windshield Defrost indicator” symbol. In accordance with previous study [29], drivers likely recognize that the malfunction defrost system may compromise driving comfort, especially in adverse weather conditions.

#### 3.6.8. Model evaluation.

The likelihood function was used to measure the goodness of fit of the model to the observed data. A lower value of -2 Log L indicates a better fit. The result indicates that the Likelihood Ratio (Chi-squared) value significantly improves its fit compared to a null model, suggesting that the predictors in the model explain a significant amount of the variance in the dependent variable (preventive maintenance compliance). The Akaike’s Information Criterion for the final model was low (AIC = 1818.946), compared to traditional ordered logistic model (AIC = 2535.637), which suggests that the model fits the data well without being overly complex, making the model more reliable and suitable for the data.

## 4. Conclusion

Vehicle dashboard indicator light symbols are an important feature for providing drivers with essential information about their vehicle’s status and functioning. The comprehension of these symbols can vary across different demographic factors. Symbols that may be clear to some drivers could be unfamiliar or misinterpreted by others. Additionally, Ghana has a unique automotive landscape influenced by the prevalence of varying vehicle models. These factors can affect how well drivers understand and respond to dashboard indicators. Furthermore, as in many other countries in Africa, Ghana relies on importation of “used” vehicles with some without vehicle manuals to aid drivers in the meaning of dashboard indicator lights, making it essential to assess the comprehensibility of these symbols among drivers. Hence, this study assesses drivers’ familiarity, and comprehension of dashboard indicator light symbols and further establishes the impact of driver background factors and comprehensibility of dashboard indicator light symbols on drivers’ preventive maintenance compliance.

The findings reveal substantial variation in drivers’ familiarity and comprehension of dashboard indicator lights symbols. While drivers were often familiar with some symbols, the percentage of them familiar with the indicator light symbols was always higher than the percentage that correctly comprehend the meaning of the symbols. The indicator light symbols that recorded higher percentage of familiarity and deem to be appropriate were comprehended by most drivers. Indicator light symbols with clear pictorial designs, such as the seatbelt and battery warning lights, were more easily comprehended, whereas symbols without these features were often misunderstood.

Interestingly, the majority of dashboard indicators that recorded low comprehension (below the 85% threshold) had a positive significant relationship with drivers’ preventive maintenance compliance. This could be that some drivers may not fully understand these indicators, but they are inclined to precautionary measures, driven by a sense of responsibility towards vehicle safety and a desire to avoid potential breakdown on the road. Hence, they are more likely to take corrective actions by inspecting and visiting a mechanic to address the issue when these indicator lights illuminate even if they do not fully understand the meaning. Even though, some drivers poorly understood some indicator lights corresponding to tyre pressure, traction, 4*4 active, power steering warning lights, they took action to comply with preventive maintenance practices. Surprisingly, even though seat belt and battery warning lights indicators are better understood (exceeded the 85% threshold), they were negatively related to drivers’ preventive maintenance compliance.

The study findings have practical implications for policymakers and various stakeholders within the automobile industry. Overall, dashboard indicator lights exhibit varying degrees of association between comprehension and drivers’ preventive maintenance compliance. The findings underline the complex relationship between comprehension of dashboard indicator lights symbols and the corrective action taken to address any related problem. One factor that can affect the familiarity and comprehension of dashboard indicator lights could be their features and attitude of drivers toward addressing issues related to indicator lights. In addition, factors such as perceived importance, urgency, and individual confidence in understanding these indicators play significant roles in determining whether action is taken or not. These findings highlight the role of understanding dashboard indicator lights play in promoting responsible safety practices among drivers. Therefore, one of the interventions is to integrate dashboard indicator light symbols into driver education programs to enhance drivers’ comprehension of the meaning and importance of these lights as well as encouraging proactive maintenance and safer driving practices. These programs can increase driver awareness and modify attitude positively and enable effective responses to dashboard warning lights. Additionally, better comprehension of indicator lights through education can equip drivers to respond to both critical and non-urgent warnings appropriately, reducing the risk of stress and promoting effective response to vehicle issues. Furthermore, manufacturers and policymakers should consider redesigning dashboard indicator symbols to make them more standardised, culturally relevant, easier to understand. Symbols that combine both pictorial and textual displays can enhance familiarity and comprehension, particularly for drivers unfamiliar with specific symbols. Revising the ISO standards for dashboard symbols to include clearer, more intuitive designs could improve communication between vehicles and drivers, reducing the risk of misinterpretation and encouraging drivers prompt action to address vehicle malfunctions.

### 4.1. Limitations and future study

The study focused on a specific set of dashboard symbols, which may limit the generalizability of its findings. Future research should consider a broader range of symbols to obtain a more comprehensive understanding of driver comprehension. Another notable limitation of this study pertains to the comprehensibility of certain symbols, particularly those associated with specific vehicle types. Different vehicle types (e.g., passenger cars, trucks, motorcycles, electric vehicles) often have unique dashboard symbols tailored to their specific functionalities. Some of these symbols may not be universally recognized or understood by all participants, especially those who are not familiar with the particular makes and models. For instance, electric vehicles (EVs) include symbols related to battery charge and regenerative braking, which may not be familiar to drivers of traditional internal combustion engine vehicles. This may introduce a potential bias in the evaluation, as participants’ familiarity with vehicle type and their corresponding symbols could influence their ability to accurately interpret the symbols. Besides, a study has also found cultural differences affecting symbol comprehension, which ties into driving environments and conditions [30]. Therefore, future studies could examine the effects of vehicle type as well as regional and cultural differences, on the familiarity and comprehension of dashboard symbols. Additionally, studies could explore how to design dashboard symbols that accommodate diverse driving populations.

Moreover, the study relied on self-reported data, which introduces potential bias as drivers may overestimate their familiarity and comprehension of dashboard indicator light symbols. Future studies could address this limitation by incorporating in-vehicle monitoring systems to observe real-time driver reactions to dashboard indicators, providing more objective and accurate data on driver behaviour and comprehension.

## Supporting information

S1 Appendix Indicator light symbols and their names. (DOCX)
