# Supplementary material for: Comprehensibility and impact of vehicle dashboard indicator light symbols on drivers’ preventive maintenance compliance
Source: PLoS One. 2025 May 14;20(5):e0323386. doi: 10.1371/journal.pone.0323386 (PMC12077779; doi:10.1371/journal.pone.0323386)
Supplement: S1 Appendix — (DOCX) [file pone.0323386.s001.docx]

**Supporting information**

**S1 Appendix.** Indicator light symbols and their names

| **S/N** | **Panel Indicator Symbol** | **Name** |
| --- | --- | --- |
|  |  |  |
| 1 | 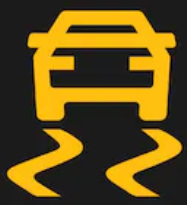 | Traction Control/ESP Light |
| 2 | 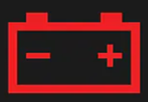 | Battery Charge Warning |
| 3 | 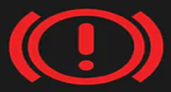 | Brake Warning Light |
| 4 | 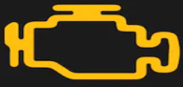 | Check Engine Light |
| 5 | 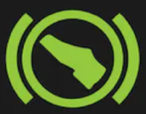 | Automatic Shift Lock |
| 6 | **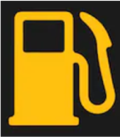** | Low Fuel Level |
| 7 | **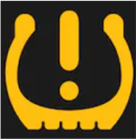** | Tire Pressure Warning Light |
| 8 | **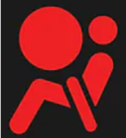** | Airbag Indicator |
| 9 | **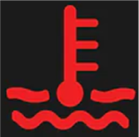** | Coolant Temperature Warning Light |
| 10 | **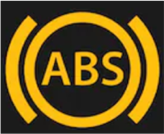** | Anti-lock Braking System |
| 11 | 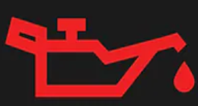 | Engine oil Pressure light |
| 12 | 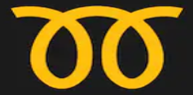 | Glow Plug Indicator (Diesel) |
| 13 | 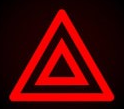 | Hazard Lights |
| 14 | 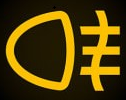 | Rear Fog Light On |
| 15 | 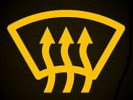 | Windshield defrost indicator light |
| 16 | 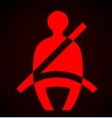 | Seatbelt Indicator |
| 17 | 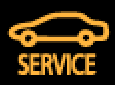 | Service Engine Soon |
| 18 | 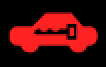 | Immobiliser Indicator |
| 19 | 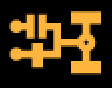 | 4*4 Gear |
| 20 | 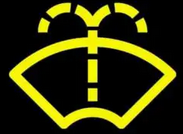 | Washer fluid warning light |
| 21 | 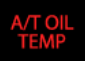 | Automatic Transmission Fluid Temperature light |
| 22 | 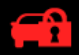 | Security Door lock |
| 23 | 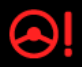 | Power Steering Warning Light |
| 24 | 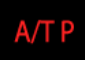 | Automatic Parking Light |
| 25 | 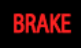 | Handbrake Engaged |
